# Vascular Risk Factors for Open Angle Glaucoma in African Eyes

**DOI:** 10.4103/0974-9233.56229

**Published:** 2009

**Authors:** Afekhide E. Omoti, Malachi E. Enock, Valentina W. Okeigbemen, Benedicta A. Akpe, Ukamaka C. Fuh

**Affiliations:** From the Department of Ophthalmology, University of Benin Teaching Hospital, P.M.B 1111, Benin City, Nigeria; 1 From the Department of Ophthalmology, Irrua Specialist Teaching Hospital, P.M.B. 08, Irrua, Edo State, Nigeria

**Keywords:** Blood Pressure, Hypertension, Open Angle Glaucoma, Perfusion Pressure

## Abstract

**Context:**

The exact patho physiological mechanizm of optic nerve damage in glaucoma is not fully understood.

**Aim:**

To assess the vascular risk factors in open angle glaucoma in African eyes.

**Settings and Design:**

Prospective multicenter hospital-based study in Edo State, Nigeria.

**Materials and Methods:**

Three hundred and twenty-two glaucoma patients and 184 control subjects were included in the study comprising 200 male glaucoma patients (62.11%) and 122 females (37.89%). A cohort of consecutive patients with a diagnosis of primary open angle glaucoma and age and sex matched control subjects were included in this prospective, cross-sectional, and multicenter hospital-based study conducted during the period January-June 2008. Blood pressure (BP), pulse and intraocular pressure findings were recorded and mean BP, pulse and perfusion pressure for each eye calculated.

**Statistical Analysis Used:**

Mean, standard deviations, 95% confidence intervals, Welch's t test, and Fisher's exact test were calculated.

**Results:**

The mean IOP in the 644 eyes of the 322 glaucoma patients was 22.65 mmHg (SD plus/minus 11.06). The systolic blood pressure (*P* < 0.0001), diastolic blood pressure (*P* < 0.0001), mean arterial blood pressure (*P* < 0.0001), pulse pressure (*P* < 0.01), systolic perfusion pressure (*P* < 0.01) were all significantly higher in glaucoma patients than in control.

**Conclusions:**

Higher systolic, diastolic, mean arterial BP and pulse pressure was found in Black African patients with open angle glaucoma.

## INTRODUCTION

The exact patho physiological mechanizm of optic nerve damage in glaucoma is not fully understood. Several vascular factors have been investigated, with particular attention to blood pressure (BP) and perfusion pressure (PP).^1^–^6^ Risk factors of vascular nature such as systemic hypertension, atherosclerosis, vasospasm, and other vascular diseases have been listed as potential factors capable of increasing the risk of OAG. According to the vascular hypothesis of open angle glaucoma (OAG) pathogenesis, a low BP relative to intraocular pressure (IOP) could lead to low PP, thus impairing perfusion of the optic nerve and causing glaucomatous loss of the visual field.^3^ On the other hand, systemic hypertension may increase risk by damaging the small vessels of the optic disc; furthermore, BP and IOP levels are positively correlated and a similar positive link between high BP and OAG might be expected.^3^ These conflicting scenarios regarding the possible effect of high or low BP levels on OAG risk have not been clarified. The relationship between risk factors such as systemic hypertension, systolic or diastolic blood pressures (BPs), or perfusion pressures and OAG remain controversial.^1^ Associations between high BP^4^–^6^ or low perfusion pressure^4^,^5^ and OAG have been reported; other studies found a relation with the ratio between BP and IOP^7^ and some others found no relation.^8^,^9^

Elucidating this relationship is important to understand the factors affecting OAG development as well as having clinical implications given the high prevalence of hypertension and use of BP-lowering treatment among older adults. The aim of this study was to examine the systolic, diastolic, mean and pulse pressure in Black African patients with OAG and compare them with the findings in control. It is not designed to determine the risk factors associated with the development or progression of the disease.

## MATERIALS AND METHODS

This is a prospective, cross-sectional, multicenter hospital-based study of some vascular risk factors in Black African patients with OAG. All patients with a diagnosis of juvenile or adult onset primary open angle glaucoma attending two tertiary hospitals, University of Benin Teaching Hospital, Benin and the Irrua Specialist Teaching Hospital, Irrua, both in Edo State, Nigeria between January and June 2008 were interviewed and examined. The diagnosis of OAG required the presence of both visual field defects and optic disc damage after ophthalmologic exclusion of other possible causes. Intraocular pressure was not considered in this definition. Visual field defects were determined by specific criteria based on automated peri metric data using the Kowa automatic visual field plotter. (Okayama, Japan). All the patients had at least two positive visual field tests. Optic disc damage was ascertained by the study of the optic discs of the patients using the Keeler specialist direct ophthalmoscope and the Volk lens with the Haag Streit slit lamp. Gonioscopy was done with the Goldmann goniolens while the intraocular pressure was measured using the Goldmann applanation tonometer. Patients were included in the study if they had:

1. Pathologically cupped optic discs- cup-to-disc ratio of 0.6 or more in association with generalized or localized thinning of the neuroretinal rim, notching of the neuro retinal rim, optic nerve head hemorrhages, nerve fiber layer loss, cup-disc asymmetry greater than 0.2, and/or deep cup with prominent lamina cribrosa and bayoneting sign.
2. Visual field defect suggestive of nerve fiber bundle defect such as arcuate scotoma in the central visual fields, nasal step, altitudinal scotoma, paracentral scotoma, and generalized field defects.
3. Open angle on gonioscopy.
4. Absence of secondary causes of glaucoma.

Patients were excluded from this study if they were on antihypertensive medication or on any drugs that may affect blood pressure or intraocular pressure. Blood pressure was assessed using the mean of two measurements in a sitting position with a mercury sphygmomanometer during one session. The patients had at least two hours rest time after arriving at the hospital before the first BP was taken and at least, another one hour before the second BP reading was taken. Systemic hypertension was defined as a systolic BP of 160 mm Hg or higher or a diastolic BP of 95 mm Hg or higher or both or the use of systemic antihypertensive medication.

The mean BP, pulse pressure and perfusion pressures for each eye were calculated.^2^ The pulse pressure was calculated as the difference between the systolic and diastolic blood pressures. The mean BP was defined as the diastolic BP plus one-third the pulse pressure. The systolic perfusion pressure was defined as the systolic blood pressure minus the intraocular pressure. The diastolic perfusion pressure was defined as the diastolic blood pressure minus the intraocular pressure while the mean perfusion pressure was the mean blood pressure minus the intraocular pressure. Age and sex matched control subjects were also examined. Those on antihypertensive medication or any drugs that may affect blood pressure were excluded. The control group was selected from patients who presented to the eye clinics with refractive errors and presbyopia.

Data analysis was done with the aid of computer using the Instat GraphPad^tm^ version 2.05a software. (San Diego, USA). The mean and standard deviations (SD) for the systolic, diastolic, mean blood pressure, pulse pressure and perfusion pressures were determined in glaucoma patients and compared with the values in control using the alternate *t* test (Welsh). The 95% confidence intervals (CI) and the standard error of the mean (SEM) were also determined. Tests for statistical significance were done using the Welch's *t* test. A *P* value of less than 0.05 was considered significant. The number of glaucoma patients with hypertension, diabetes mellitus and migraine were compared to the number of control subjects with the same conditions using the Fisher's exact test and the odds ratio calculated.

Ethical approval was obtained from the ethical committee of the University of Benin Teaching Hospital, Benin City, Nigeria.

## RESULTS

A total of 3472 patients in the two hospitals were screened; 322 consecutive glaucoma patients who met the inclusion criteria and 184 control subjects were included in the study. There were 200 male glaucoma patients (62.11%) and 122 females (37.89%). The mean age was 55.91 years (SD plus/minus 18.31). The SEM was 1.443 and the 95% CI was 53.08-58.74. The median age was 61 years. The age and sex distribution of the glaucoma patients is shown in Table 1 while the age and sex distribution of the control is shown in Table 2.

**Table 1:** Age and sex distribution of patients with open angle glaucoma

Age (years)	Male (%)	Female (%)	Total(%)
10-19	14 (4.35)	10 (3.10)	24 (7.45)
20-29	14 (4.35)	4 (1.24)	18 (5.59)
30-39	14 (4.35)	4 (1.24)	18 (5.59)
40-49	18 (5.59)	14 (4.35)	32 (9.94)
50-59	30 (9.32)	26 (8.07)	56 (17.39)
60-69	58 (18.01)	34 (10.56)	92 (28.57)
70 and Above	54 (16.77)	28 (8.70)	82 (25.47)
Total	200 (62.11)	122 (37.89)	322 (100)

**Table 2:** Age and sex distribution of control subjects

Age (years)	Male (%)	Female (%)	Total (%)
10-19	8 (4.3)	6 (3.3)	14 (7.6)
20-29	8 (4.3)	3 (1.6)	11 (5.9)
30-39	12 (6.5)	4 (2.2)	16 (8.7)
40-49	10 (5.4)	7 (3.8)	17 (9.2)
50-59	18 (9.8)	13 (7.1)	31 (16.9)
60-69	36 (19.6)	19 (10.3)	55 (29.9)
70 and above	25 (13.7)	15 (8.1)	40 (21.8)
Total	117 (63.6)	67 (36.4)	184 (100)

The mean IOP in the 644 eyes of the 322 glaucoma patients was 22.65 mmHg (SD plus/minus 11.06) and the SEM was 0.80. The range was 9-68mmHg and the 95% CI was 20.92-24.37. All the glaucoma patients had features of glaucoma in both eyes. The patients were not classified according to severity of glaucoma, The mean IOP in the 368 eyes of 184 control subjects was 13.47 mmHg (SD plus/minus 3.23) and the SEM was 0.17. The range was 7-23mmHg and the 95% CI was 13.14-13.80. The Welch's approximate *t* is equal to 19.648, df is equal to 817 and the two-tailed *P* value was less than 0.0001 which was statistically significant.

The vascular factors are shown in Table 3. History of smoking was not evaluated in this study. The systolic BP (*t* is equal to 7.363, df is equal to 461, *P* less than 0.0001), diastolic blood pressure (*t* is equal to 9.040, df is equal to 494, *P less than* 0.0001), mean arterial blood pressure (*t* is equal to 6.316, df is equal to 372, *P less than* 0.0001), pulse pressure (*t* is equal to 2.904, df is equal to 364, *P less than* 0.01), systolic perfusion pressure (*t* is equal to 2.978, df is equal to 459, *P less than* 0.01) were all significantly higher in glaucoma patients than in control. There was no significant difference in diastolic perfusion pressure, mean perfusion pressure and pulse rate between glaucoma patients and control (*P* greater than 0.05). A total of 118 patients (36.65%) with glaucoma were found to have hypertension while six subjects (3.26%) out of the control were found to have hypertension (*P less than* 0.0001, Fisher's exact test). The odds ratio was 17.160 (95% CI is equal to 7.373-39.937). Twenty-six patients (8.01%) with glaucoma had diabetes mellitus while two subjects (1.01%) out of the control had diabetes (*P is equal* to 0.0004, Fisher's exact test). The odds ratio was 7.993 (95% CI is equal to 1.874-34.689). There was no glaucoma patient or control subject that had a past medical history of an event resulting in vascular collapse. Six glaucoma patients (1.86%) had migraine while four control subjects (2.17%) had migraine (*P is equal* to 1.0000, Fisher's exact test). The odds ratio was 0.8544 (95% CI is equal to 0.2379-3.069). This was not statistically significant.

**Table 3:** Vascular risk factors in glaucoma and control subjects

Vascular factors	Glaucoma Mean ± SD	Patients 95% CI	Control Mean ± SD	Subjects 95% CI	Welch's *t*	Degree of freedom	*P* value
Systolic blood pressure (mmHg)	135.85 ± 25.57	133.06 - 138.64	20 ± 19.70	118.02 - 1 23.72	7.363	461	<0.0001
Diastolic blood pressure (mmHg)	83.88 ± 16.09	82.12 - 85.64	73.15 ± 10.55	71.63 - 74.67	9.040	494	<0.0001
Mean arterial blood pressure (mmHg)	102.40 ± 18.64	100.36 - 104.44	91.33 ± 19.15	88.56 - 94.10	6.316	372	<0.0001
Pulse pressure (mmHg)	52.18 ± 15.67	50.47 - 58.89	47.83 ± 16.51	45.44 - 50.22	2.904	364	0.0039
Systolic perfusion pressure (mmHg)	113.51 ± 26.14	110.65 - 116.37	107.30 ± 20.24	104.38 - 110.22	2.978	459	0.0031
Diastolic perfusion pressure (mmHg)	61.04 ± 19.37	58.92 - 63.16	60.00 ± 11.67	58.31 - 61.69	0.7534	502	0.4515
Mean perfusion pressure (mmHg)	79.41 ± 21.33	77.08 - 81.78	77.43 ± 18.78	74.72 - 80.94	1.085	421	0.2785
Pulse rate (No/minute)	75.30 ± 9.33	74.28 - 76.32	76 ± 7.26	74.95 - 77.05	0.9381	458	0.3487

## DISCUSSION

This study was designed simply to compare the vascular risk factors in POAG with control. It was not designed to discover new risk factors or determine the risk of developing glaucoma. A vascular role in the patho physiologic mechanism of optic nerve damage in OAG has been studied extensively.^1^,^3^–^5^ Despite the biologic rationale for a causal link between these vascular risk factors and glaucoma, the epidemiologic evidence is inconsistent and difficult to interpret.^10^ Several conflicting reports have been published. In this study there was a significantly higher incidence of hypertension among the glaucoma patients compared with control. This may be related to selection bias which exists in hospital based studies. Hypertension is also a risk factor for POAG.^11^ This may also contribute to the significant association of hypertension and OAG in this study. Epidemiologic reviews reflect conflicting data regarding the role of systemic hypertension in glaucoma, which never seemed to make much physiologic sense.^12^ If the vascular supply of the optic nerve and the ganglion cells play a role in the glaucomatous process, then in early glaucoma, when the ‘head of pressure’ is high, hypertension should if anything, be protective if one ignores the role of auto regulation of vascular resistance and resultant blood flow.^12^ Late in uncontrolled hypertension, vascular sclerosis should reduce blood flow despite an elevated BP, thereby increasing the risk of glaucomatous damage.^12^,^13^ Like many previous studies, the Baltimore Eye Survey only showed a modest positive association was noted between systolic and diastolic BP and primary open angle glaucoma.^11^ However, when age was corrected for, a stronger association was then found among the elderly.

The Barbados Eye Study^2^,^3^,^14^ which was carried out on a large population of Black people showed that the incidence of glaucoma was high in this African-descent population, where the established factors of older age, higher IOP, and family history contributed to risk. Additional predictors were vascular factors, including lower systolic BP, and particularly lower ocular perfusion pressures, which more than doubled the risk. Thinner central corneal thickness was also a factor. These findings indicate a multifactorial etiology of open angle glaucoma.^2^ Although hypertension and diabetes were common in Barbados Eye Study participants, they were unrelated to the prevalence of open-angle glaucoma.^14^ Persons with systemic hypertension at baseline had half the RR, suggesting that hypertension does not increase (and may decrease) the 4-year risk of open angle glaucoma.^3^ The differences in the finding of this report and the Barbados Eye Studies may be related to the study population and design. This study was a hospital based study and included patients from the second to the ninth decades of life while the Barbados Eye Study was a population based study which included patients from the fifth to the ninth decades of life. All the patients in this study had features of glaucoma in both eyes although their presentation may be asymmetrical. This is because late presentation in open angle glaucoma is frequent in Nigeria with patients presenting in the advanced stages of the disease.^15^ By this time the disease is usually bilateral. In this study, systolic, diastolic, mean arterial, pulse and systolic perfusion pressure were all significantly higher in glaucoma patients than in controls. This may be due to the significantly higher incidence of hypertension among glaucoma patients. Contrary to expectations, there was no significant difference in diastolic perfusion pressure, mean perfusion pressure and pulse rate between glaucoma patients and control subjects. This may be because this study analysed all age groups together and the fact that, the BP measured at the arm may not be fully representative of the blood flow in the small blood vessels of the eye in elderly hypertensive patients due to the secondary vascular sclerotic changes. Thus, these elderly hypertensive patients may actually have a reduced blood flow at the vascular capillaries and therefore reduced perfusion pressure. More refined measures of vascular perfusion, applied to individuals of different age and with different structural characteristics of their optic nerve, might well provide important clues to identifying those at greatest risk.

Considerable attention has been paid to hemodynamic crises and related dramatic drops in blood pressure, particularly as a potential causative factor in normal tension glaucoma.^12^ However, there was no history suggestive of dramatic drop in blood pressure in any of the patients in this study including those with normal tension glaucoma. However lack of this history is not proof of absence of previous hemodynamic crisis. Despite the rapid rise in risk of glaucoma with diastolic perfusion pressures below 50 mmHg, less than one-third of all glaucoma patients fall within this category.^11^ The actual physiology is undoubtedly more complex, given the intricacies of perfusion pressure, auto regulation of vascular resistance, and other parameters we cannot yet measure.^12^

Diabetes was also found to be significantly higher in patients with OAG in this study. This is in agreement with previous studies which also report this association.^16^,^17^ There are however a number of studies that show no association.^18^–^20^ The Beaver Dam study concluded that primary open-angle glaucoma was twice as common among older-onset diabetes than among non diabetic patients.^17^ The Baltimore eye survey on the other hand failed to provide confirmation that diabetes was a risk factor for glaucoma but provided clues as to how this belief may have arisen.^18^ In their study, diabetes was not associated with primary open-angle glaucoma but patients whose glaucoma had been diagnosed before the examination showed a positive association with diabetes indicating that selection bias could explain the positive results of previous clinic-based studies.

In conclusion, hypertension and diabetes were more common among patients with OAG than in control in Black Africans. The systolic, diastolic, mean arterial and pulse pressures were significantly higher in glaucoma patients. A lower perfusion pressure could not be demonstrated in this study. A long term follow-up study of healthy subjects who later develop OAG may be able to associate lower perfusion pressure with glaucoma and other vascular risk factors not readily apparent in this study.
